# A first look into moss living tardigrades in boreal peatlands

**DOI:** 10.1002/ece3.70045

**Published:** 2024-08-01

**Authors:** Hennariikka Mäenpää, Merja Elo, Sara Calhim

**Affiliations:** ^1^ Department of Biological and Environmental Science University of Jyväskylä Jyväskylä Finland; ^2^ Nature Solutions Unit Finnish Environment Institute Jyväskylä Finland

**Keywords:** community ecology, habitat heterogeneity, micrometazoans, Tardigrada

## Abstract

Tardigrades (Tardigrada) are a phylum of micrometazoans found in all biomes on Earth, but their ecology and habitat preferences remain vastly understudied. Boreal peatlands include a diversity of habitat types and high structural heterogeneity that represents an interesting system to study some of the poorly known habitat preferences of tardigrades. Here, we investigate for the first time tardigrade communities in peatland mosses and the latter's potential associations with key environmental variables. We collected 116 moss samples from 13 sites representing different peatland types and management histories. We found that tardigrades are common and diverse in boreal peatlands, as tardigrades were present in 72% of the collected samples and we identified 14 tardigrade genera. Tardigrade abundance seemed to increase alongside the increasing tree basal area and the density was higher in the microtopographic level further from the water table level, that is, hummocks (mean 117/moss gram) than in lawns/hollows (mean 84/moss gram). Furthermore, the highest tardigrade density was found in the moss taxa that are associated with forested peatland types (i.e., feather mosses) (321 mean/moss gram). Finally, we found interesting patterns regarding tardigrade functional diversity, as carnivorous tardigrades were found only in peatlands with tree basal area > 20 m^2^ and mostly in hummocks. Our study demonstrates that the habitat heterogeneity of peatlands (e.g., variation in moisture and vegetation cover) represents an interesting system to study tardigrade ecology and habitat preferences. However, since we found variation in tardigrade abundance and communities across peatland types and microhabitats within peatlands, our results highlight that such studies should be conducted with numerous replicate samples and a systematic study design that properly addresses the habitat heterogeneity between and within different peatland types.

## Graphical abstract

We investigate for the first time tardigrade community composition in boreal peatlands. Tardigrades are common in boreal peatlands and their abundance and community composition varies across peatland habitat types and microhabitats within peatlands.

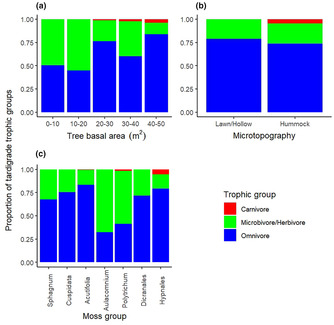

## 1 INTRODUCTION

Tardigrades are micrometazoans that exist in all biomes on Earth in marine, freshwater, and terrestrial habitats (Nelson et al., 2015). Of the over 1400 tardigrade species described (Degma & Guidetti, 2023), most live in terrestrial habitats in moist substrates such as soil, mosses, and lichens (Bartels et al., 2016; Møbjerg et al., 2018; Nelson et al., 2018). Tardigrade taxa represent different trophic groups: herbivorous species that feed mainly on algae, microbivores that feed on microbes, carnivores that feed on other micrometazoans and other tardigrades, and omnivores with a mixed diet (Nelson, 2002; Tůmová et al., 2022). Tardigrades are mostly known for their ability to survive extreme conditions by undergoing a reversible dormant state, cryptobiosis, which allows them to withstand extreme temperatures, drying, osmotic pressure, changes in salinity, or oxygen deficiency (Guidetti & Møbjerg, 2018; Hengherr & Schill, 2018; Møbjerg & Neves, 2021; Schill & Hengherr, 2018). However, to stay active and reproduce in terrestrial environments, they need to be surrounded by a film of water (Møbjerg et al., 2018). The ability to undergo cryptobiosis in all life stages has allowed tardigrades to colonize variety of environments and limnoterrestrial tardigrades are often found in high densities in substrates that experience contrasting wet and dry periods (Nelson et al., 2015).

Despite the increasing number of studies on, for example, tardigrade cell biology (Jönsson et al., 2019), dormancy strategies (Møbjerg & Neves, 2021), taxonomy (Fleming & Arakawa, 2021), and reproductive behavior (Chartrain et al., 2023; Sugiura & Matsumoto, 2021), tardigrade ecology and their habitat preferences remain vastly understudied (Nelson et al., 2018). Spatial variation in tardigrade occurrence, that is, patchiness, is a common feature for tardigrade populations (Nelson, 2002) and variation in occurrence has been found even within moss cushions (Degma et al., 2011). Studies on the effects of environmental factors have included, for example, rainfall, humidity, and temperature (Degma et al., 2011; Jönsson, 2007; Nelson & Bartels, 2013; Schuster & Greven, 2007), substrate type (Guil & Sanchez‐Moreno, 2013; Jönsson, 2003; Kathman & Cross, 1991; Nelson et al., 2020), altitude (Guil et al., 2009; Kathman & Cross, 1991), and anthropogenic stress (Jönsson, 2003; Nelson et al., 2020). However, the results of the studies have often been contrasting and the impact of different environmental variables on tardigrade occurrence remains unclear (discussed e.g., in Nelson et al., 2020). It has been discussed whether the spatial variation in occurrence and population densities is a consequence of passive distribution, carried out by wind, rain, and floodwaters or more determined by habitat conditions. However, many studies have concluded that favorable conditions for the population growth of tardigrades arise from combinations of macro‐, meso‐, and microscale environmental factors (Guidetti et al., 2024; Guil et al., 2009).

As moist and moss‐rich habitats, boreal peatlands could be ideal habitats for tardigrades. Yet, they have been completely overlooked in tardigrade studies. This is probably because peatlands are often acidic, a condition to which tardigrades are known to be vulnerable (Massa et al., 2023). However, the variation in acidity among and even within peatland ecosystems is notable; the pH in ombrotrophic bogs can be below 4, whereas in rich fens it may be between 5 and 7 (Rydin et al., 2013; Tahvanainen, 2004). In addition to spatial variation, the peatland water pH may vary seasonally and even the diurnal variation may be 0.5–1 pH units (Tahvanainen & Tuomaala, 2003). Boreal peatlands are complex ecosystems that include a diversity of habitat types and high structural heterogeneity (Kareksela et al., 2021; Rydin et al., 2013). Numerous species rely on peatland habitats and many species are specialized in certain peatland habitat types, especially among invertebrates (Hannigan & Kelly‐Quinn, 2012; Hyvärinen et al., 2019).

The habitat heterogeneity between and within peatland types represents an interesting system to study the poorly known habitat preferences of tardigrades. The definition of a peatland differs across countries, but it usually refers to a peat‐accumulating ecosystem with peat depth of at least 30 cm (Rydin et al., 2013). Some peatlands receive water and nutrients solely from precipitation and airborne dust (ombrotrophic), whereas some are nourished by mineral‐soil‐influenced groundwater (minerotrophic). Additionally, peatlands are classified from poor to high‐level productivity ecosystems as oligotrophic, mesotrophic, or eutrophic. These features affect the peatland water chemistry (e.g., pH) and together with water availability influence the species composition of plant communities (Tahvanainen, 2004), soil invertebrates (Batzer et al., 2016), and microorganisms (Rydin et al., 2013). Consequently, peatland habitat types vary from poor nutrient and low productivity level treeless fens that are mostly characterized by *Sphagnum* mosses and low pH to rich fens and wooded mires with higher productivity and more diverse vegetation. From the perspective of small invertebrates, such as tardigrades, the spatial heterogeneity within peatlands arises from combinations of different scale environmental variables that are created by the physical structure of vegetation and water availability (Hannigan & Kelly‐Quinn, 2012). Macroscale environmental variables affecting heterogeneity in habitat conditions are, for example, tree cover, shrubs, sedges, and grasses that affect the exposure to sunlight at the surface. Mesoscale variation is created by the microtopography of peatlands (hummock, lawn, hollow, pool) that creates habitats with different moisture levels (Rydin et al., 2013). The higher levels of the microtopography, that is, hummocks, are further from the water table, and, therefore, seasonal changes in water availability may be notable in these microhabitats. By contrast, the lower levels, lawns, and hollows, provide habitats with more continuous water availability and pools are constantly covered by water. The surface vegetation consists mostly of mosses and adds microscale habitat heterogeneity that arises from moss species‐specific characteristics, such as different growth forms of mosses.

In this paper, we present the very first study of tardigrade communities in boreal peatland mosses. We aim to (1) describe the abundance and diversity of tardigrade communities, and (2) assess changes in tardigrade abundance and communities among environmental variables at the macro‐ (tree cover), meso‐ (microtopographic level), and microscales (moss type).

## 2 METHODS

### 2.1 Study sites

**FIGURE 1:**
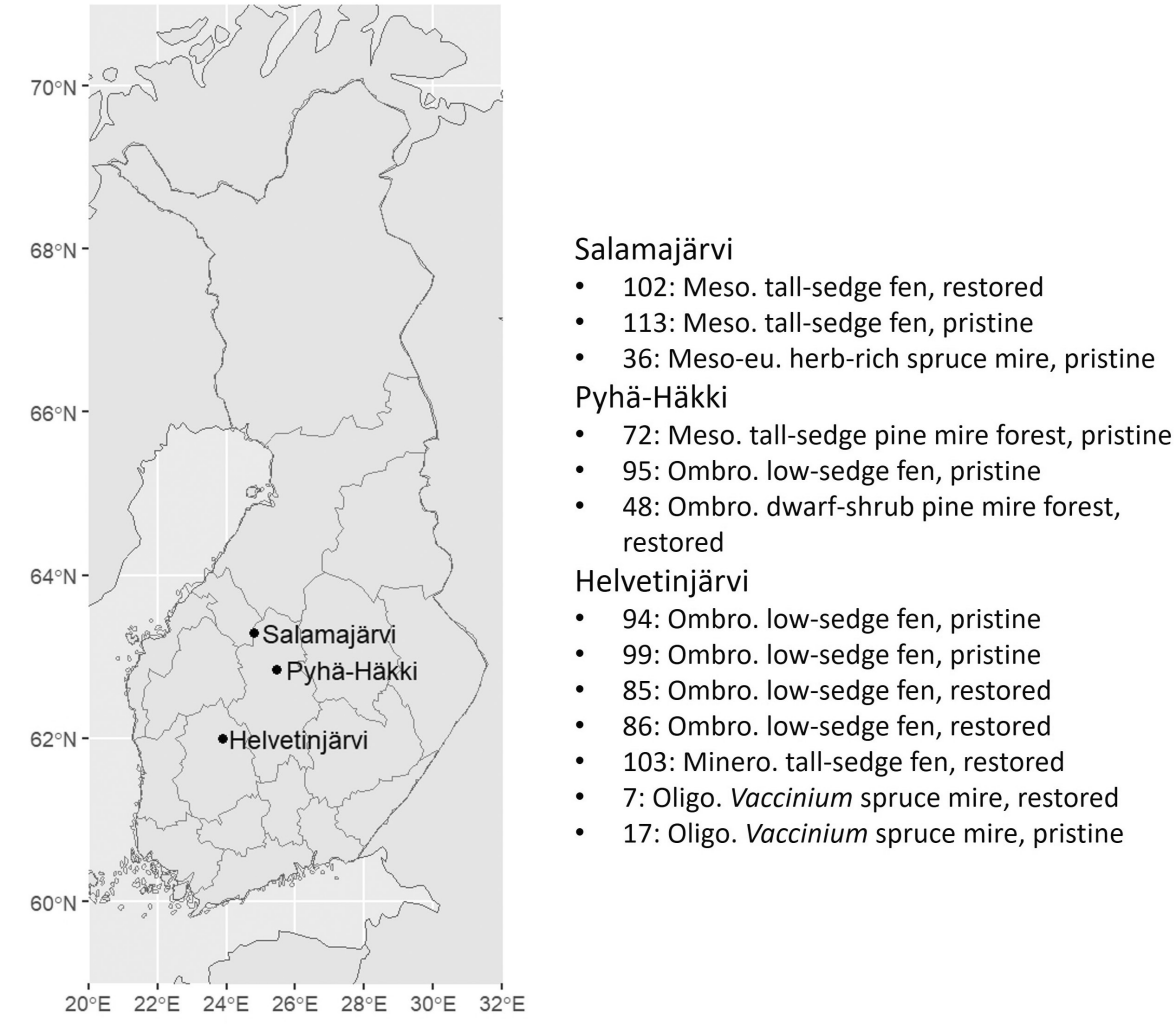
The locations of the three national parks where the sampled peatland sites are situated. The sites are listed according to their site ID in the peatland restoration network and vegetation types and management histories. English translations of the peatland vegetation types are according to Kontula and Raunio (2019). The geospatial data for the map were downloaded from Statistics Finland (retrieved in April 2024).

We sampled a total of 13 peatland sites located in Salamajärvi, Pyhä‐Häkki, and Helvetinjärvi National parks near Central Finland (Figure 1) in September 2020. Since this was the very first investigation on what kind of boreal peatland habitats tardigrades may occur, these sites represent a range of habitat types and management histories. Eight sites are open fens (four pristine and four restored), two pine mire forests (one pristine and one restored), and three spruce mire forests (two pristine and one restored).

Our study sites are part of the peatland restoration monitoring network that was established by Metsähallitus Parks & Wildlife Finland and the University of Jyväskylä in 2007 (Elo et al., 2024). The peatland restoration network has been a subject of research for over a decade including studies on the effects of drainage and restoration on, for example, peatland hydrology, vegetation, invertebrates, and bird communities (Alsila et al., 2021; Elo et al., 2015; Haapalehto et al., 2014; Maanavilja et al., 2014).

### 2.2 Description of the peatland types used in this study

The detailed vegetation types of the sampled peatlands are listed in Figure 1 and in Data S1.

Fens are either completely treeless or grow sparsely in some stunted pine trees, mostly *Pinus sylvestris* and the surface is dominated by *Sphagnum* mosses (e.g., *S*. *papillosum* and *S*. *balticum*). In ombrotrophic fens vascular plants consist mostly of patches of low‐sedges (e.g., *Eriophorum vaginatum*), whereas the surface in minerotrophic fens has a higher coverage of brown mosses and diversity and abundance of vascular plants, such as tall‐sedges (e.g., *Carex lasiocarpa*) and grasses (e.g., *Equisetum fluviatile*). The peat layer in fens may be several meters deep, the water table is slightly below or just above the surface and the microtopography is formed by lawns, hollows, pools, and some low hummocks (Kaakinen et al., 2018; Rydin et al., 2013).

Pine mire forests are wooded peatlands that grow mostly *P*. *sylvestris* but especially in rich types some sparsely growing Norway spruce (*Picea abies*) and birch (*Betula pubescens*) may occur. *Sphagnum* mosses dominate the lower and more moist levels of the surface microtopography (such as *S*. *fallax*, *S*. *angustifolium*, *S*. *divinum*, *S*. *Magellanicum*, and *S*. *russowii*) and patches of forest mosses (e.g., *Pleurozium schreberi*) and *Dicranum* mosses grow especially on hummocks and tree bases. In ombrotrophic pine mire forests the understory vegetation is characterized by densely growing dwarf shrubs (*Rhododendron tomentosum*, *Chamaedaphne calyculata*, *Betula nana*) and sedges (e.g., *Carex pauciflora*a and *Carex globularis*), whereas mesotrophic pine mire forests grow more tall‐sedges (e.g., *Carex lasiocarpa*, *Eriophorum vaginatum*). The peat layer is often deep (1–1.5 m), the water table level is below the surface and the seasonal variation in moisture levels may be notable (Eurola et al., 2015; Kaakinen et al., 2018).

Spruce mire forests have typically a dense cover of Norway spruce but also birch and other deciduous trees (e.g., *Populus tremula*) are common, especially in rich types. The water table is normally well below the surface but small‐scale variation in microtopography and moisture is typical. Spruce mire forests are often situated in the ecotones of mineral soil forests and peatlands and species composition has characteristics of both. Consequently, they are highly diverse, mosaic‐like habitats. The ecosystem is classified as a peatland if over half of the area is covered by peatland vegetation and peat layer is more than 20 cm deep (Eurola et al., 2015). Species that are more typical for peatland habitats, such as *Sphagnum* mosses (*S*. *girgensohnii*, *S*. *russowii*, *S*. *squarrosum*, and *S*. *centrale*) occur in depressions that are continuously wet, while forest mosses (e.g., *Hylocomnium splendens*, *Polytrichum commune*) are common on drier surfaces. Understory vegetation consists mostly of shrubs (e.g., *Salix* spp., *Juniperus communis*), heaths (e.g., *Vaccinium myrtillus*, *V*. *vitis‐idaea*), forest grasses (e.g., *Equisetum sylvaticum*, *Maianthemum bifolium*), and sedges (e.g., *C*. *globularis*) (Kaakinen et al., 2018).

During the time of our sampling, the mean annual precipitation at the sites was 0.01 mm and the mean annual temperature varied between 5.4 and 6.2°C from North to South, respectively.

The restored peatland sites had been drained for forestry purposes during 1960s and 1970s to decrease the water table level and increase timber production. The restoration of the peatlands took place in 2010 and 2011 and included filling or blocking the ditches to raise the water table and enable the natural functions and structures of the ecosystems, for example, hydrology, community composition, and cycling and fluxes of nutrients.

### 2.3 Sampling

We collected a total of 116 moss samples from all peatland types from the immediate proximity of the permanent vegetation study plots in the peatlands (see more detailed description of the study sites in Elo et al., 2016). Samples consisted of roughly a handful (c. 10 cm diameter) of a single moss species that were identified in the field and confirmed in the laboratory using a dissecting microscope (Olympus SZX9, 10× magnification). In addition, we documented the microtopographic level where the sample was collected from (hummock or lawn/hollow).

We stored the moss samples in 1 L sealable plastic bags and placed them into coolers to prevent the temperature from increasing and avoid tardigrades dying inside the bags. The samples were transported to the University of Jyväskylä where we extracted tardigrades from the moss samples immediately after transportation by using the Baermann wet funnel method with sieves of 1 mm mesh size and 24 h extraction duration (described in more detail in Mäenpää et al., 2023). We took random parts of moss from each sample and covered the 10 cm diameter sieves with them (one sample/sieve) and stored the remaining sample. We placed the sieves in the funnel cones and covered the moss entirely with tap water. After the extraction, the moss samples were collected from the sieves and dried at 60°C for 48 h and weighed at precision of 1 mg (Mettler Toledo XS204 DeltaRange). We counted the tardigrades from each sample under a dissecting microscope (Olympus SZX9, 10× magnification). Because of the high number of individuals, we mounted a maximum of randomly selected 70 specimens from each sample on slides in Hoyer's medium for identification. We chose this number to control for the effort that is needed for identification and to reach at least 20% identification success of the total number of individuals collected. We identified the specimens to genus level under a phase contrast microscope (Zeiss AXIO, 100× magnification) by using updated taxonomic accounts and descriptions. A list of the genera found in each sample is provided in Data S1 and a figure of proportions of the genera across the levels of environmental variables can be found in Data S2.

### 2.4 Data

To estimate the density of tardigrades in a standardized amount of substrate, we divided the number of tardigrades by the dry weight of moss (g) that was placed in the funnels. The number of identified specimens in each genus was calculated in relation to the total number of tardigrades found in the samples. We classified the identified genera into three trophic groups they represent: carnivorous, herbivorous/microbivorous, and omnivorous. The classification was done based on the dimensions of the buccal‐pharyngeal apparatus of the identified genera (Guidetti et al., 2012; Tůmová et al., 2022). The classification was similar to what was done in Zawierucha et al. (2019), with the exception that we classified the genus *Milnesium* as carnivorous since it can reproduce only with animal prey in its diet (Bryndová et al., 2020). A list of tardigrade genera and the trophic groups can be found in Data S1.

We used tree basal area of the sites (macroscale), microtopographic location of the samples (mesoscale), and moss type in the samples (microscale) as different scale environmental variables to describe the variation in habitat conditions. The data for the tree basal area were measured from a circular area around the vegetation study plots with a radius of 10 m and were collected by Metsähallitus Parks & Wildlife Finland. The tree basal area at the sites varied between 0 and 50 m^2^ and we used it as a five‐level factor that was grouped by 10 m^2^. The microtopographic location of the samples was included as a two‐level factor hummock or lawn/carpet, pools were not sampled. We grouped the moss species into seven groups that describe their substrate characteristics. *Sphagnum* species were grouped by subgenus: *Sphagnum* (large sized, robust, dense cushions), *Cuspidata* (median sized, forms loose mats), and *Acutifolia* (small to median sized, dense cushions or tussocks). *Dicranales*, *Aulacomnium*, and *Polytrichum* were grouped by genus and Hypnales order mosses (feather mosses). The data are provided in Data S3.

We used R version 4.2.1 (R Core Team, 2022) and the R‐packages ggplot2 (Wickham, 2016) and dplyr (Wickham et al., 2023). The geospatial data for the map in Figure 1 were downloaded with the R‐package geofi (Kainu et al., 2023).

## 3 RESULTS AND DISCUSSION

### 3.1 Tardigrade abundance in the studied peatlands

We investigated the associations between macro‐, meso‐, and microscale environmental variables and the community composition of moss living tardigrades in boreal peatlands in Finland. More specifically, we compared the variation in mean abundance, community trophic structure, and taxonomic diversity (family) in relation to tree basal area of the sites, microtopographic location, and sample moss type (Table 1). Tardigrades were present in 72% of the 116 samples that included a total of 7546 tardigrades, of which 1436 specimens were identified successfully into 14 tardigrade genera within eight families. The density of tardigrades across all samples varied between 0 and 1228 specimens/moss gram and the overall mean was 72 specimens/moss gram. Based on our findings, tardigrades are common in peatland mosses and their abundance can be high in some peatland habitats and microhabitat types. As a comparison, we found on average a higher total number of tardigrades in our samples than what has been found in some former studies in other habitat and substrate types: Guil et al. (2009) found a total of 11,019 individuals from 288 moss and leaf litter samples, Degma et al. (2011) found a total of 224 individuals in 25 samples of a single moss species growing on a rock, Guil and Sanchez‐Moreno (2013) found a total of 3303 tardigrade individuals from 144 leaf litter samples, and Nelson et al. (2020) found a total of 900 individuals from 336 moss and lichen samples collected from tree trunks. Note, however, that the total number of tardigrades found across different studies could also be affected by other factors than habitat/substrate types, such as the volume of collected substrate per sample (not always reported) or the extraction methods used (discussed, e.g., in Czerneková et al., 2018, Mäenpää et al., 2023).

**TABLE 1:** A list of the macro‐, meso‐, and microlevel environmental factors including the peatland type where samples were collected from, the proportion of samples that included tardigrades, and the mean of tardigrades per moss gram ± standard error of the mean.

Tree basal area	Peatland type(s)	Samples with tardigrades/Total number of samples	Mean of tardigrade number/moss gram (±SD)
0–10	Open fen	26/50	14 (±25)
10–20	Open fen (r), Pine mire forest	10/17	33 (±35)
20–30	Pine mire forest	8/19	66 (±60)
30–40	Open fen (r), Spruce mire forest	19/19	131 (±196)
40–50	Spruce mire forest	20/21	248 (±339)
*Microtopography*			
Lawn/Hollow	All types sampled	33/43	84 (±156)
Hummock	All types sampled	50/73	117 (±240)
*Moss genus*			
*Sphagnum*	All types sampled	9/24	42 (±65)
*Acutifolia*	All types sampled	19/29	43 (±61)
*Cuspidata*	All types sampled	24/31	75 (±177)
*Polytrichum*	Open fen (r), Open fen, Pine mire forest, Spruce mire forest	9/10	23 (±18)
*Aulacomnium*	Open fen (r), Pine mire forest	3/3	53 (±42)
*Dicranales*	Pine mire forest (r), Open fen (r), Spruce mire forest (r)	3/3	47 (±32)
*Hypnales*	Open fen (r), Pine mire forest (r), Pine mire forest, Spruce mire forest	16/16	321 (±351)

*Note*: The mean of tardigrades/moss gram is calculated based on the samples that had tardigrades. (r) after peatland type refers to restored peatland.

The 28% of our samples that did not have tardigrades were mostly collected from treeless ombrotrophic bogs and oligotrophic fens (Table 1). If tardigrades were present, the densities were low, between 1 and 84 individuals per moss gram. This could be expected since many tardigrade species are known to be sensitive to acidic (pH <5) conditions (Massa et al., 2023) and low oxygen levels (Møbjerg & Neves, 2021) which are common in these peatland types. However, these conditions may vary spatially and temporally within peatlands (Tahvanainen & Tuomaala, 2003), which may explain tardigrade occurrence despite the expected overall unfavorable conditions. The samples that included 60% of all tardigrades we found were collected from the two *Vaccinium* spruce mire forests with a tree basal area > 40 m^2^. Mosaic‐like small‐scale variations in surface microtopography and diverse understory vegetation are characteristic for spruce mire forest and create high heterogeneity in microhabitat conditions. Habitat heterogeneity generally affects the species richness and, for instance, high spatial heterogeneity is important for the diversity in assemblages of aquatic macroinvertebrates in peatlands (Josué et al., 2015). In addition, the dense canopy cover provides shelter from solar radiation and enables cool, humid, and stable microclimatic conditions. Consequently, spruce mire forests are typically species‐rich ecosystems and are often referred as biodiversity hotspots in boreal forest landscapes (Kaakinen et al., 2018). It is likely that the spatial habitat heterogeneity and stable microclimate also provide a large range of suitable habitat conditions for tardigrades and the relatively cool and stable microclimate may enable population growth, as some tardigrade species may be sensitive to changes in temperature (Neves et al., 2022; Schuster & Greven, 2007). As a comparison, spatial and temporal changes in surface temperature may be notable in treeless peatlands and may vary greatly even between neighboring patches of different moss species in otherwise seemingly similar conditions (Leonard et al., 2021; Stoy et al., 2012).

### 3.2 Macroscale patterns – Tree basal area

**FIGURE 2:**
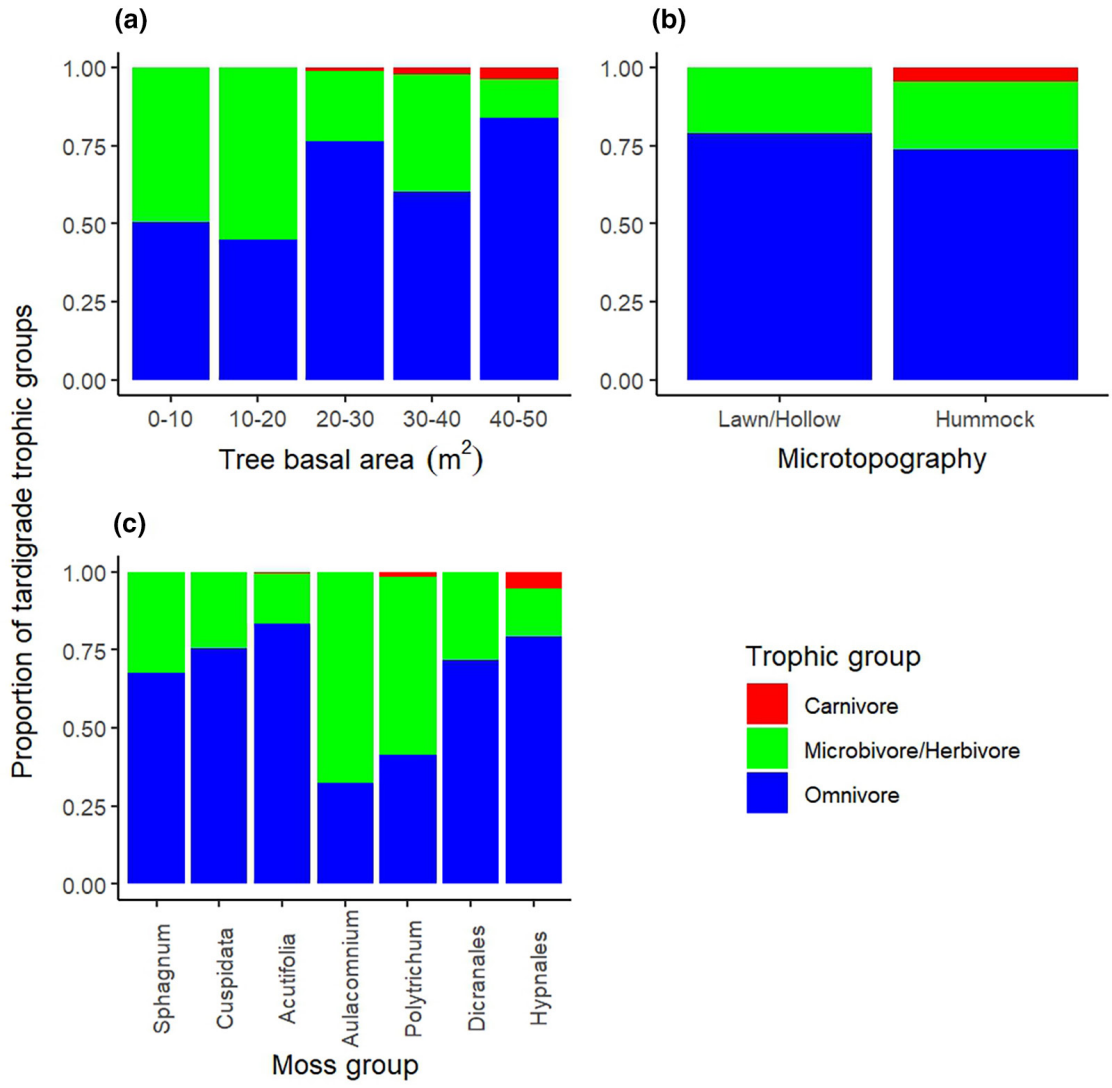
The proportion of the three trophic groups of tardigrades in relation to tree basal area (m^2^), the microtopographic location of the sample, and the moss group. The proportions of the trophic groups are calculated based on the identified specimens in relation to the total number of tardigrades found in the samples. The tree basal area was measured within 10 m radius around the sampling sites.

**FIGURE 3:**
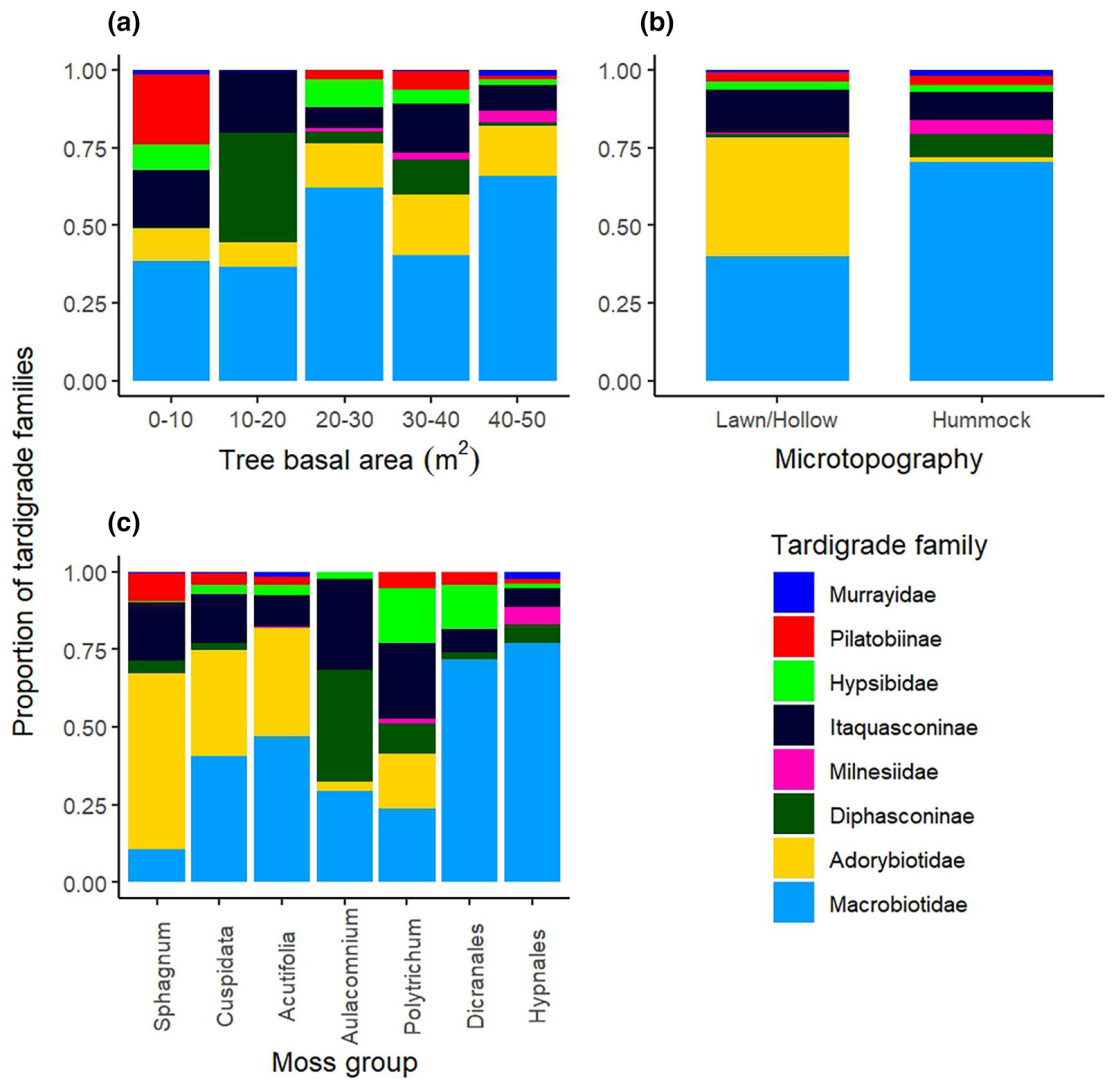
The proportion of tardigrades in each family in relation to tree basal area (m^2^), microtopographic location of the sample, and moss group. The proportions of tardigrade families are calculated as trophic groups in Figure 1 and the tree basal area is measured in Figure 1.

Generally, tardigrade density and the diversity of communities seemed to increase with the degree of tree cover (i.e., tree basal area) of the studied peatlands (Table 1). Carnivorous tardigrades (family Milnesiidae) were found only in peatlands with tree basal area > 20 m^2^. However, even in these habitats, the proportion of carnivorous tardigrades remained relatively low (2–5%) (Figure 2). Omnivorous tardigrades seemed to be the dominant trophic group, except in peatlands with tree basal area < 20 m^2^ where the proportion of microbivorous/herbivorous taxa was equal or slightly higher (Figure 2), and the overall abundance of tardigrades was low (Table 1). The microbivorous/herbivorous tardigrades found in these less wooded peatlands included families that also occurred in more wooded peatland types, albeit in lower proportions (Figure 3). This may be partly explained by the absence of carnivorous tardigrades, as they may prey on many smaller tardigrades (e.g., *Hypsibius* and *Diphascon*) (Nelson, 2002). Another explanation for the low abundance of tardigrades and higher proportion of microbivorous/herbivorous taxa in open peatlands could be the availability of resources. All peatland types host relatively diverse communities of microbes, such as bacteria, fungi, and algae, and the composition of these communities depends on moisture, oxygen and nutrient levels, acidity (Jaatinen et al., 2007; Rydin et al., 2013) and the vegetation community (Littlewood et al., 2010). The microbial communities of peatlands, especially microalgae and fungi, follow the rich‐poor nutrient gradient, as observed for vascular plants (Rydin et al., 2013). It is possible that the microbial communities in more wet‐ and nutrient‐poor open peatlands provide sufficient food resources for relatively small populations of microbivorous/herbivorous tardigrades to persist. However, the more diverse microbial communities of nutrient‐rich wooded peatlands can support larger/more diverse communities of micrometazoans, such as the increased abundance and diversity in trophic groups reported for nematodes (Ferris et al., 2001; Kamath et al., 2022). Hence, it is likely that more diverse microbial communities also enable more abundant and diverse tardigrade communities to exist through trophic group interactions. Further understanding of these interactions and the consequences to tardigrade communities would require estimating the possible food resources and predators of tardigrades (e.g., prokaryotes, fungi, algae, rotifers, nematodes, mites, flatworms).

### 3.3 Mesoscale patterns—Microtopography (hummock vs. lawn/hollow)

Peatlands with higher tree basal area generally have a lower water table level that in turn enables tree growth. Moreover, the microtopography of wooded peatlands is dominated by relatively dry hummocks that are also likely to experience high variations in moisture depending on climatic conditions. The variation in water table level across and within peatlands is one of the key elements defining habitat suitability, especially for small invertebrates. For example, the community composition of testate amoebae is mainly impacted by peatland hydrology across different peatland types (Beaulne et al., 2018; Daza Secco et al., 2016) and moisture regimes are the key determinant of nematode communities in peatlands (Kamath et al., 2022; Wang et al., 2021; Wei et al., 2018). Interestingly, we found higher tardigrade abundance in hummocks compared to lawns/hollows that provide more continuous water availability (Table 1). In addition, most of the carnivorous tardigrades were also found from hummocks (Figure 2). It has been commonly noted that even though terrestrial tardigrades require a film of water to stay active, the highest population densities and species richness are typically found in habitats with alternate periods of wet and dry conditions (Nelson et al., 2015). Similar patterns have also been found in studies on tardigrades in rock pools: higher tardigrade population density (Jocqué et al., 2007) and more diverse communities (Vecchi et al., 2022) have been found in rockpools that have shorter wet periods and are in risk of periodic desiccation. Experimentally increased hydration has also been found to have species‐specific responses on tardigrade population densities in mosses growing on rocks, including a negative impact on predator taxa (Jönsson, 2007). In some studies, moisture conditions have shown to be irrelevant for tardigrade occurrence in mosses and not to impact either their vertical (Nelson & Adkins, 2001) or horizontal (Degma et al., 2011) distribution within moss cushions. In contrast, a recent study by Guidetti et al. (2024) found a positive correlation between species richness of tardigrades and increased rainfall in mosses in a study across boreal forests in different regions of Norway. Altogether, studies have shown contrasting results of the connection between moisture and tardigrade occurrence, and it is likely to affect species differently depending on, for example, their anhydrobiosis ability. Moreover, studies addressing the correlation between moisture and tardigrade occurrence have been conducted across a variety of substrate and habitat types, which may partly explain the contrasting results, as the impact of moisture may vary among these and affect other habitat properties. For instance, soil pH is affected by increased moisture (Slessarev et al., 2016; Zarate‐Valdez et al., 2006), rainfall water chemical properties may depend on canopy cover quantity and quality (Zhao et al., 2023), and water retention capacity varies in different substrates (e.g., moss species) (Glime, 2007).

### 3.4 Microscale patterns—Moss group

As in some previous studies (Guidetti et al., 2024; Jönsson, 2003; Mäenpää et al., 2023), we found (Table 1) the highest tardigrade densities in the moss group Hypnales (the feather mosses *Pleurozium schreberi* and *Hylocomnium splendens*), which are commonly found in the hummocks of more wooded peatland types. Over 80% of the identified specimens in Hypnales mosses were omnivorous, of which most belonged to the family Macrobiotidae (Figures 2 and 3). These mosses also included the highest proportion of carnivorous tardigrades, that is, the family Milnesidae (5%). The most notable exception to the dominance of family Macrobiotidae was the high proportion of the family Adorybiotidae (genus *Crenubiotus*) in all subgroups of *Sphagnum* mosses and in lawns/hollows. Interestingly, the proportion of family Adorybiotidae seemed slightly higher in *Sphagnum* mosses collected from forested peatlands. Even though *Sphagnum* mosses are characteristic for treeless and less nutrient‐rich peatlands, none are restricted to poor peatlands but rather are the only ones that can thrive in those conditions. Many *Sphagnum* species exist in all types of peatlands, whereas some can survive only in more nutrient‐rich peatlands and are therefore used as indicator species of the peatland habitat types (Eurola et al., 2015). In forested peatlands, *Sphagnum* mosses can be typically found in the lower and more wet levels of the microtopography. So far, no species‐specific correlations between tardigrades and their substrates have been found in the few studies that have attempted to address the question (e.g., Kaczmarek et al., 2011; Nelson et al., 2020). However, some differences in tardigrade abundance and species richness have been linked to growth form of mosses, to large‐scale environmental factors such as forest type, or to the microscale habitat conditions within mosses (Guidetti et al., 2024; Jönsson, 2003). The growth form of the mosses affects, for example, their water retention capacity (Glime, 2007) and temperature within the moss cushion (Stoy et al., 2012). However, more detailed substrate information (e.g., taxonomic groups or growth forms of mosses/lichens) is rarely included in the analysis on tardigrade distribution and would add valuable information on the microenvironment level effects on tardigrade ecology. In addition to microscale climatic conditions, especially feather mosses often host a wide range of algae, cyanobacteria, and fungi that tardigrades feed on and that are affected by large‐scale environmental variables, such as climate and canopy cover (Kauserud et al., 2008; Renaudin et al., 2022).

### 3.5 Implications for future studies

In general, our observations in peatland mosses align with earlier conclusions that tardigrade distribution is likely influenced by combinations of different scale environmental variables, that in turn determine the overall habitat conditions within substrates (Guidetti et al., 2024; Guil et al., 2009; Jönsson, 2003). Note, however, that the data we present reflects a random, yet uneven, sampling across a wide range of peatland habitat types with different levels of anthropogenic disturbance. The number and the size of the moss substrate sampled varied between sites, which could affect the probability of finding tardigrades, considering the patchiness of their occurrence. Therefore, our observations are merely descriptive. Moreover, since we identified tardigrades only to genus level, we cannot know whether there is species‐specific variation across the levels of the included environmental variables within speciose groups such as the family Macrobiotidae.

Although these data preclude formal statistical analyses, our findings nonetheless provide an important first look at what factors may affect tardigrade communities in an understudied key ecosystem–boreal peatlands. First, it is evident that assessing the differences between peatland habitat types requires a large sample size with numerous within habitat type replicates. Second, to further understand the effects of the fine‐scale habitat heterogeneity within peatlands, a systematic sampling design that encompasses different levels of the microtopography within peatlands is needed and, if possible, one that includes information on the substrate characteristics (e.g., moss growth form). An interesting addition would be to estimate the quantity of other microscopic fauna that may affect tardigrade distribution through predation or competition. Large‐scale ecological studies on tardigrades are laborious, and processing (i.e., extracting, counting, and mounting tardigrades) numerous (i.e., hundreds) samples is highly time‐consuming. The latter is probably the main reason for the rarity of sufficient data in most studies (discussed, e.g., in Nelson et al., 2018). Despite the extensive research effort, sufficient sample size with replicate samples across large‐scale (e.g., habitat type) and small‐scale (e.g., microhabitat conditions) environmental variables is fundamental to further understand tardigrade ecology. Third, when studying ecosystems that have been subjected to considerable anthropogenic disturbance, such as drained and restored peatlands, the management history of the study sites should be considered. Lastly, to ensure the accuracy of identification, an integrative approach should be used by combining molecular and morphological characters, especially when identifying to species level (see, e.g., Topstad et al., 2021). Identifying tardigrades to species level based solely on morphology is challenging partly because of the phenotypical plasticity within species and because it often requires collecting and identifying eggs, which is not always possible. While determining species level variation across different habitat conditions is important for faunistic studies and understanding species‐specific adaptations, incorrect species identification may cause misleading interpretations of tardigrade diversity and distribution (discussed in, e.g., Momeni et al., 2023). Therefore, in studies on tardigrade ecology, decisions on methodology (morphological and/or molecular analysis) and the sufficient taxonomic level of identification for the aims of the research in question should be considered carefully.

## 4 CONCLUSIONS

We conclude that tardigrades are common in boreal peatland habitats with variation in abundance and community composition across different peatland habitat types and between microhabitats within peatlands. It is likely that this variation arises from combinations of different macro‐, meso‐, and microscale environmental variables that determine the microhabitat conditions in mosses. However, to further understand this variation, large‐scale systematic sampling with replicate samples across different peatland habitat types and microtopography within peatlands is needed. Altogether, our findings show that the habitat heterogeneity of peatlands provides an interesting system for further studies on some of the poorly known aspects of tardigrade ecology and habitat preferences.

## AUTHOR CONTRIBUTIONS

**Hennariikka Mäenpää:** Conceptualization (equal); data curation (lead); formal analysis (lead); funding acquisition (equal); investigation (lead); methodology (equal); resources (equal); software (equal); validation (equal); visualization (lead); writing – original draft (lead). **Merja Elo:** Conceptualization (equal); data curation (supporting); formal analysis (supporting); investigation (supporting); methodology (equal); project administration (supporting); software (equal); supervision (equal); validation (equal); visualization (supporting); writing – original draft (supporting); writing – review and editing (equal). **Sara Calhim:** Conceptualization (equal); data curation (supporting); formal analysis (supporting); funding acquisition (equal); investigation (supporting); methodology (equal); project administration (lead); resources (equal); software (equal); supervision (equal); validation (equal); visualization (supporting); writing – original draft (supporting); writing – review and editing (equal).

## CONFLICT OF INTEREST STATEMENT

All authors declare that they have no conflicts of interest.

## Supporting information


Data S1:

Data S2:

Data S3:

## Data Availability

Data are available in the supplementary material of the article.
