# Supplementary material for: A first look into moss living tardigrades in boreal peatlands
Source: Ecol Evol. 2024 Aug 1;14(8):e70045. doi: 10.1002/ece3.70045 (PMC11293882; doi:10.1002/ece3.70045)
Supplement: Supplementary file 2 — Data S2: [file ECE3-14-e70045-s003.docx]

Supplemetary material to Mäenpää H., Elo M., and Calhim S. A first look into moss living tardigrades in boreal peatlands.


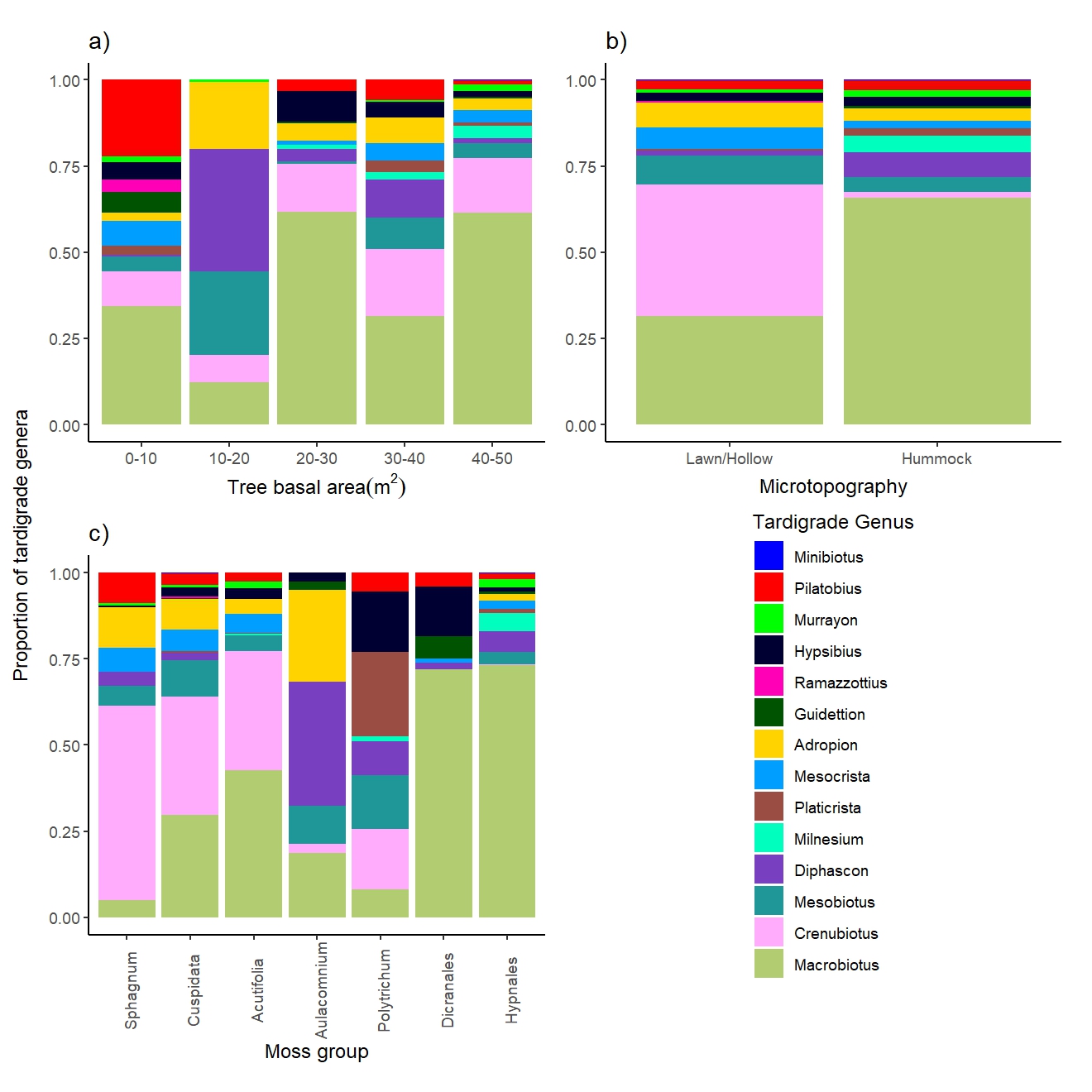


Figure S1. The proportion of tardigrade genera in relation to tree basal area (m^2^), microtopographic location of the sample and moss type. The tree basal area was measured within 10 m radius around the sampling sites. The proportion of the genera is calculated based on identified specimens in relation to the total number of tardigrades found in the samples.
